# SPE-IMS-MS: An automated platform for sub-sixty second surveillance of endogenous metabolites and xenobiotics in biofluids

**DOI:** 10.1016/j.clinms.2016.11.002

**Published:** 2016-12-29

**Authors:** Xing Zhang, Michelle Romm, Xueyun Zheng, Erika M. Zink, Young-Mo Kim, Kristin E. Burnum-Johnson, Daniel J. Orton, Alex Apffel, Yehia M. Ibrahim, Matthew E. Monroe, Ronald J. Moore, Jordan N. Smith, Jian Ma, Ryan S. Renslow, Dennis G. Thomas, Anne E. Blackwell, Glenn Swinford, John Sausen, Ruwan T. Kurulugama, Nathan Eno, Ed Darland, George Stafford, John Fjeldsted, Thomas O. Metz, Justin G. Teeguarden, Richard D. Smith, Erin S. Baker

**Affiliations:** a Earth and Biological Sciences Division, Pacific Northwest National Laboratory, Richland, WA, United States; b Department of Environmental and Molecular Toxicology, Oregon State University, Corvallis, OR, United States; c Agilent Technologies, Santa Clara, CA, United States

**Keywords:** Ion mobility spectrometry, Mass spectrometry, Metabolomics, Exposomics

## Abstract

Characterization of endogenous metabolites and xenobiotics is essential to deconvoluting the genetic and environmental causes of disease. However, surveillance of chemical exposure and disease-related changes in large cohorts requires an analytical platform that offers rapid measurement, high sensitivity, efficient separation, broad dynamic range, and application to an expansive chemical space. Here, we present a novel platform for small molecule analyses that addresses these requirements by combining solid-phase extraction with ion mobility spectrometry and mass spectrometry (SPE-IMS-MS). This platform is capable of performing both targeted and global measurements of endogenous metabolites and xenobiotics in human biofluids with high reproducibility (CV 6 3%), sensitivity (LODs in the pM range in biofluids) and throughput (10-s sample-to-sample duty cycle). We report application of this platform to the analysis of human urine from patients with and without type 1 diabetes, where we observed statistically significant variations in the concentration of disaccharides and previously unreported chemical isomers. This SPE-IMS-MS platform overcomes many of the current challenges of large-scale metabolomic and exposomic analyses and offers a viable option for population and patient cohort screening in an effort to gain insights into disease processes and human environmental chemical exposure.

## 1. Introduction

The identification and detection of endogenous metabolites and xenobiotics has evolved over the past decade to encompass metabolic pathway characterization, disease diagnosis [1], metabolic profiling [2], biomarker discovery [3], and environmental exposure assessment [4]. While the evaluation of endogenous metabolites has played an important role in understanding naturally occurring biochemical pathways and bodily changes, measurements of xenobiotics, such as drugs and environmental contaminants, are also important. Following completion of the human genome project, the role of xenobiotics was further highlighted as >90% of human diseases were not unilaterally determined based on genetic profile, but involved a combination of genetic and environmental factors [5–8]. Thus, recent small molecule metabolomic and exposomic analyses have been directed at characterizing endogenous molecules as well as environmental chemical exposures [8–10]. The term exposome was introduced in 2005 to describe how exposure to natural chemicals from bacteria, plants, and animals, and synthetic molecules in food, water, air, drugs, and consumer products interact with endogenous chemicals in the body [8]. The comprehensive characterization of exposure, however, is difficult since exposure levels are estimated to be on average, greater than one thousand chemicals per day per person and occur from a structurally and physico-chemically diverse group of molecules [8]. Analysis of these chemicals can also prove difficult given their varying range of concentrations (i.e., femtomolar to millimolar) and matrix of analysis (e.g., plasma, urine and saliva). Further, to fully understand the relationship between chemical exposure and human disease, samples from a large cohort of individuals are required. Thus, development of analytical instrumentation with improved throughput and separation efficiency and less dependence on sample preparation and derivatization will be essential for future metabolomic and exposomic studies.

Gas chromatography–mass spectrometry (GC–MS) [11,12] and liquid chromatography–mass spectrometry (LC–MS) [13–15] are the most widely used analytical platforms for small molecule analyses. Each platform has important strengths including high sensitivity and multi-dimensional molecular characterization; however, when applied to comprehensive sample characterization, their modest throughput (i.e., ~1–2 samples per hour) limits their ability to process large cohorts in a reasonable timeframe. While the high feature resolution of GC–MS has led to significant contributions in small molecule studies, it has limited utility for large-scale analyses due to its requirement for chemical derivatization of poorly volatile compounds, propensity for interference by relatively high-abundance substances (e.g., sugars), and modest sample throughput. For LC–MS, the chemical diversity of small molecules typically requires the use of several LC column packing materials and corresponding mobile phases in order to accommodate the diverse chemical classes, which presents a major barrier to comprehensive analysis in a single run. Furthermore, the need for different ionization sources and positive and negative ionization modes to detect the range of chemicals being screened is a challenge for all MS methods. Approaches such as high-throughput screening matrix assisted laser desorption ionization (MALDI)-MS [16], LC-MALDI-MS [17], fast salt purges using fused silica capillary with resin [18], and rapid capillary electrophoresis on microwell plates for whole-plate sample preparation [19] have demonstrated promise for some rapid analyses, but normally take more than one minute and have been challenging to automate for routine studies.

The integration of ion mobility spectrometry (IMS) with MS has emerged as an appealing approach for high-throughput small molecule analyses, offering structural and mass separation for each endogenous metabolite or xenobiotic in a single run [20]. IMS allows rapid, high-resolution separation of chemicals during passage through a drift cell containing a buffer gas, which is typically nitrogen or helium. IMS separations do not require derivatization and can be combined with different ionization sources for analysis of distinct compound types, such as polar metabolites using electrospray ionization (ESI) in positive or negative ionization modes, and nonpolar compounds, such as polyaromatic hydrocarbons using atmospheric pressure chemical ionization (APCI) or atmospheric pressure photoionization (APPI) in positive or negative ionization modes. Additionally, IMS-MS can separate chemical isomers [21–27] and takes less than one second per analysis even when multiple spectra are summed. However, ion suppression, due to the presence of highly abundant chemicals, has limited the utility of IMS-MS in untargeted analyses of complex mixtures. Here, we report what we believe to be the first combination of a unique, high throughput SPE system [28–30] coupled with IMS-MS that solves many of the challenges facing large-scale analyses of endogenous metabolites and xenobiotics by providing measurements with high sensitivity, high-throughput, absolute target quantitation, effective separation of isomeric compounds, applicability to a broad chemical space with little modification, and multidimensional information for chemical identification. The utility of this platform is demonstrated using human plasma and urine samples, and further illustrated with a case-control study of individuals affected by type 1 diabetes (T1D).

## 2. Materials & methods

### 2.1. Standard sample preparation

All standards and reagents were purchased from Sigma-Aldrich (St. Louis, MO, USA). Human plasma samples were also purchased from Sigma and stored at −80 °C until further use. 500 μL of ice-cold acetonitrile was added to 50 μL of each plasma sample, and vigorously mixed for 1 min. After equilibration for 10 min at room temperature, the samples were centrifuged at 12,000g at 4 °C for 20 min. The supernatants containing extracted metabolites were collected for SPE-IMS-MS analysis. An aliquot of the human plasma was run at least once every 20 samples and used as quality control for all spiked plasma measurements.

Human urine standard samples were purchased from BioreclamationIVT and stored at −80 °C until further use. A urease pre-treatment procedure was performed before methanol extraction on the urine samples. 50 μL of urease solution was added to 50 μL of urine samples, vortexed briefly and spun down. The mixture was incubated at 37 °C with mild shaking (500 rpm) for 30 min and then cooled on ice for 1 min. Methanol extraction was performed by adding 1 mL of ice-cold methanol to the urine solution after urease pre-treatment. The mixture was then vortexed for 30s and centrifuged at 15,000g at 4°C for 10 min. The supernatants were collected and dried under speed vacuum for 4 h. 500 μL HPLC grade water was added for SPE-IMS-MS analyses. An aliquot of the human urine was run at least once every 20 samples and used as quality control for all urine measurements.

Twenty xenobiotic standards (Table S1) were analyzed individually at concentration levels of 10 pM, 100 pM, 1 nM, 10 nM, 100 nM and 1 μM to determine their limits of detection (LODs) prior to biofluid addition. A mixed stock solution was then prepared using a 99.9/0.1 acetonitrile/formic acid buffer solution and 10 μM of each chemical for SPE-IMS-MS method optimization. The mix was added into the human plasma extract and human urine extract to achieve final concentration levels of 500 pM, 1 nM, 5 nM, 10 nM, 50 nM and 100 nM for each chemical and the LOD was determined in both biofluids (Table S1).

### 2.2. Human Subjects, urine sample collection, and processing urine specimens

Urine was collected from T1D patients and healthy siblings associated with Children’s National Medical Center (CNMC). The Childhood and Adolescent Diabetes program at the CNMC is the largest center in the mid-Atlantic region of the United States, caring for approximately 1600 juvenile patients with T1D. A comprehensive human subject protocol was developed to ensure patient confidentiality, including all clinical and molecular meta-data collected during recruitment, and that the procedures for specimen collection were not associated with direct health risks. A human subject consent form was designed with sufficient information for the to-be-enrolled sibling pairs and their parents/guardians (for subjects less than 18 years of age) to allow them an assessment of the public benefits of the research and the confidentiality loss or health risks. The consent form and protocol were approved by the Internal Review Boards at Pacific Northwest National Laboratory (PNNL) and CNMC. PNNL staff had access to patient identifier codes but not the names or addresses of the study participants. The urine specimens were stored up to 4 weeks at −20 °C at the CNMC clinical site and transferred to the PNNL for storage at −80 °C until further use. The urine was then prepared in the same way as indicated above for the human urine standard samples.

### 2.3. Instrumentation

A RapidFire 365 Solid Phase Extraction (SPE) (Agilent Technologies, Santa Clara, CA) was interfaced to an Agilent 6560 Ion Mobility QTOF MS system [31,32] (Agilent Technologies, Santa Clara, CA). The RapidFire 365 uses high-speed robotics and fast-switching valves to facilitate ultra-fast online SPE. A 10 μL sample loop was used for all experiments. A C18 SPE cartridge (Agilent Technologies, Santa Clara, CA) was selected for the initial experiments. The system was optimized to maximize the binding efficiency, while minimizing carry-over, using the xenobiotic standard mixture. To overload the sample loop, 30 μL of each urine sample was loaded onto the SPE cartridge using a combination of 0.1% formic acid and 99.9% water (v/v) (mobile phase A, flow rate at 1.5 mL/min), and eluted using a combination of 0.1% formic acid, 49.95% isopropanol (IPA) and 49.95% methanol (v/v/v) (mobile phase B, flow rate at 0.6 mL/min). Based on C18 SPE cartridge optimization of the small molecule polar extracts from urine, mixed mode, graphitic carbon, and HILIC cartridges were used to maximize detection coverage of small molecules with differing affinities. The same mobile phase A and B were applied to the mixed mode cartridge as described for the C18 cartridge. For the graphitic carbon cartridge, the polar urine extracts were loaded using the same mobile phase A as used for C18 cartridge, but the small molecules were eluted using a combination of 0.1% formic acid, 25% acetonitrile, 25% acetone and 49.9% water (v/v/v/v) (mobile phase B, flow rate at 0.6 mL/min). For the HILIC cartridge, the polar urine extracts were loaded using a combination of 0.1% formic acid, 10% IPA, 89.9% hexane (v/v/v) (mobile phase A, flow rate at 1.5 mL/min), and small molecules were eluted using the same mobile phase B as used for the C18 cartridge. Aspiration time, load time, elution time and re-equilibration time were 0.6 s, 3 s, 6 s, and 1 ms, respectively, for a total cycle time of ~10 s. The sample injection sipper and valve loops were washed with water followed by acetonitrile between each sample injection.

The Agilent 6560 used in this work couples a drift tube ion mobility spectrometer to a quadrupole time-of-flight mass spectrometer. A jet stream orthogonal electrospray ionization source was utilized to connect the RapidFire with the IMS-QTOF MS. Ions generated by ESI were transmitted through the glass capillary, high-pressure ion funnel and the ion funnel trap (IFT). The IFT pulsed ions into the IMS drift tube (78 cm) filled with nitrogen, where a uniform electric field (16.5 V/cm) was applied and pressure was maintained at 3.95 Torr. Ions were separated in the drift tube and refocused by a rear ion funnel before entering the Q-TOF MS. Additional instrumentation details can be found elsewhere [31,32].

## 3. Results & discussion

### 3.1. IMS-MS separation of endogenous metabolite and xenobiotic isomers

**Fig. 1:**
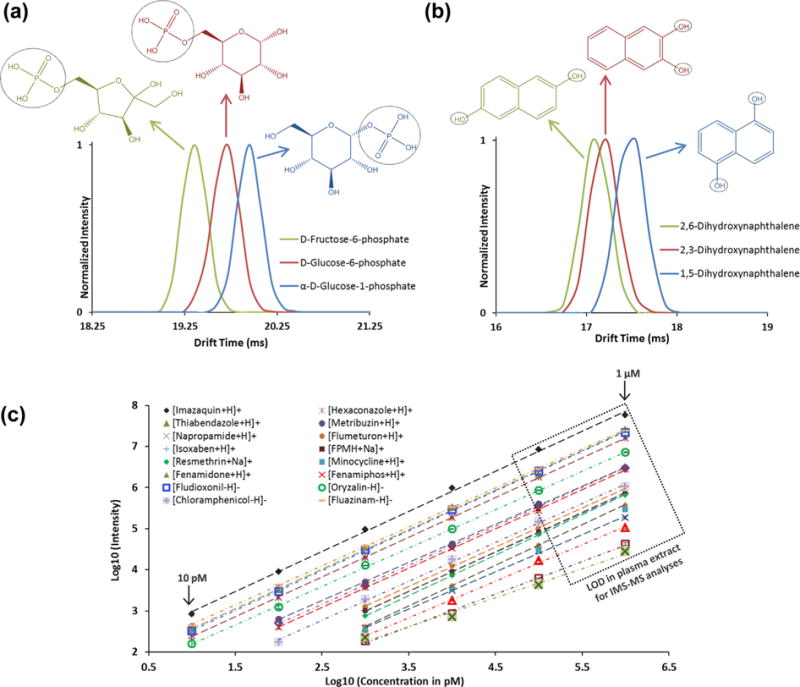
IMS spectra for the deprotonated isomers of a) fructose-6-phosphate, glucose-6-phoshate, and glucose-1-phosphate and b) 2,6-dihydroxynaphthalene, 2,3-dihydroxynaphthalene and 1,5-dihydroxynaphthalene. Each isomer was separated in the IMS dimension, but was unresolvable by MS due to the isomers having the same mass. c) A 6-point calibration curve for 20 xenobiotics analyzed individually in water/methanol with varying concentrations (10 pM, 100 pM, 1 nM, 10 nM, 100 nM, and 1 μM). Error bars from triplicate analyses are included for each point and the box indicates the LOD of each compound spiked into a plasma extract and analyzed with IMS-MS alone.

A major limitation of MS analysis is the inability to distinguish isomers and ions with similar *m/z* values, especially since many small molecules have identical or nearly indistinguishable empirical formulas. A classic example involves fructose-6-phosphate, glucose-6-phosphate, and glucose-1-phosphate, which are all endogenous metabolites of importance in glucose metabolism. These metabolites have the same mass and empirical formula, differing only in their structural arrangement. Fig. 1a demonstrates the resolving power of IMS-MS in separation of these compounds where fructose-6-phosphate has the smallest structure (as indicated by the shortest drift time) and glucose-1-phosphate the largest (longest drift time).

Similarly, IMS-MS was used to resolve three xenobiotic dihydroxynaphthalene isomers having different hydroxyl placements (2,6; 2,3; and 1,5). As shown in Fig. 1b, IMS-MS easily distinguished 1,5-dihydroxynaphthalene from the other two isomers, but 2,6 and 2,3-dihydroxynaphthalene had similar drift times. 2,6-dihydroxynaphthalene, however, was observed to have the smallest structure of the three isomers. Computational modeling can be performed to understand the ordering of these isomers, as shown previously [23,27]. These isomer analyses illustrate the ability of IMS to distinguish both endogenous metabolites and xenobiotics that are not separable with MS alone, and, with a per-sample analysis time of less than one second, facilitate high-throughput, small molecule analysis.

### 3.2. High throughput SPE separations increase sensitivity in complex biological matrices

A common challenge associated with complex sample analyses is ion suppression and matrix interference, both of which result in poor sensitivity and inadequate LODs. To evaluate the IMS-MS analysis of endogenous metabolites and xenobiotics in complex biological matrices, the LOD and calibration curve linearity of 20 environmental chemicals were compared, after being spiked into water, urine, and plasma. Prior to these spiking experiments, baseline studies were performed on the water, urine, and plasma to ensure that none of the 20 xenobiotics were present at detectable levels, and that there were no detectable interferences.

The individual xenobiotic standards, spiked into water, showed an LOD of ~10 pM and highly linear calibration curves from 10 pM to 1 μM (coefficient of determination [r^2^] ≥ 0.99) (Fig. 1c). In comparison, when spiked into urine and plasma, the LODs for the same chemicals increased from 10 pM to high nM levels (Fig. 1c), indicating ion suppression and/or interference from salt and/or matrix, which frequently occurs and hinders analysis of complex biological samples [33]. These observations illustrate the need for a clean-up or separation step prior to the IMS-MS analyses. To address this challenge, while preserving conditions for high-throughput analysis, we explored the use of rapid solid-phase extractions (SPE).

**Fig. 2:**
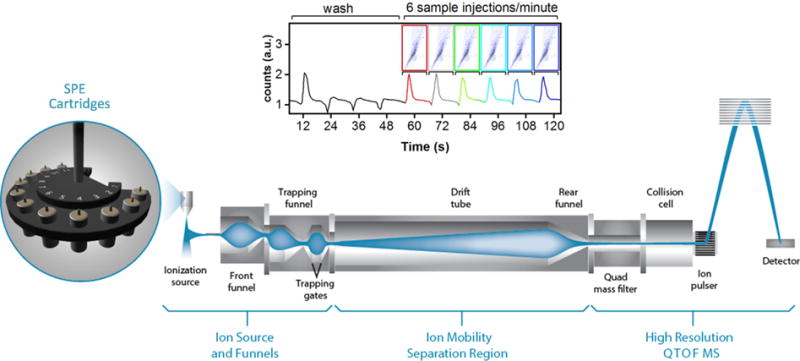
The workflow for the sub-sixty second small molecule analyses using the automated SPE system coupled with an IMS-QTOF MS system. Multiple SPE cartridges can be used for each sample to analyze chemicals with distinct characteristics. After SPE separation, the sample stream is ionized and the resulting ions travel through the IMS separation region for structure separation, followed by detection using high resolution QTOF MS to achieve accurate *m/z* measurements. Each SPE-IMS-MS analysis was optimized to have a 10-s sample-to-sample duty cycle as shown by the 6 urine injections at the top of the figure.

Automated SPE systems, which utilize reusable, microscale SPE cartridges of varying packing composition, have been successfully combined with MS for rapid, front-end separations, followed by sensitive, targeted chemical analyses [28,34,35]. These automated SPE systems provide a 5- to10-s sample-to-sample duty cycle, which is 2 to 3 orders of magnitude faster than conventional GC or LC methods (Fig. 2). While the automated online SPE systems may not be as thorough as offline (and slower) SPE methods for removing all contaminates, they enable rapid analysis times that can be utilized in a high-throughput pipeline. While the results for SPE-MS targeted analyses have been promising, its utility for both targeted and untargeted studies with complex samples has been limited since many isomers bind to the same cartridge matrix, thereby hindering confident identification by MS alone. However, when coupled with IMS-MS, SPE provides benefits for both targeted and discovery metabolomic studies, especially when analyzing complicated biological matrices, such as biofluids (Fig. 2).

Targeted SPE analyses are normally performed by optimizing a single SPE cartridge for high-throughput screening of a limited number of chemical species. In global studies, multiple SPE cartridges (*e.g.*, mixed mode, graphitic carbon and/or HILIC cartridges) can be utilized to detect an expanded range of small molecules. Each cartridge is selected to achieve maximum binding of the specific molecular class of interest, while removing potentially interfering components present in the biological sample that exhibit low binding affinity to the packing material in the cartridge.

**Fig. 3:**
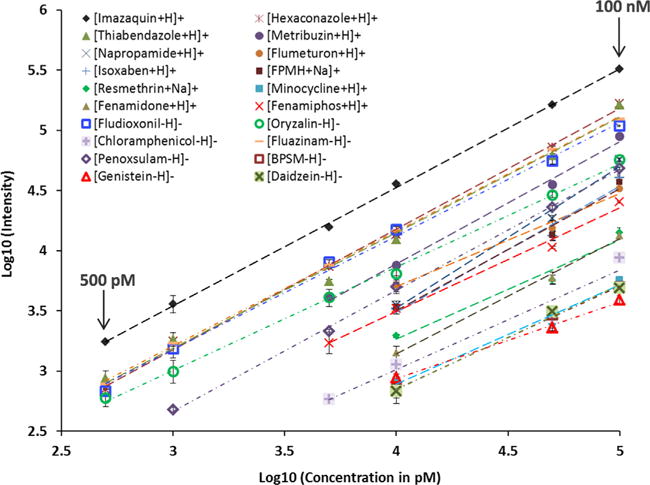
Six-point calibration curve for 20 xenobiotics spiked into a human plasma with varying concentrations (500 pM, 1 nM, 5 nM, 10 nM, 50 nM, and 100 nM). The average CV of three replicates is reflected in the error bars.

The increase in IMS-MS sensitivity obtained from the SPE front-end separation was quantitatively evaluated by comparing the LODs and calibration curves for the 20 xenobiotics spiked into blood and urine with and without front-end separation. The 20 calibration curves shown in Fig. 3 illustrate that all 20 xenobiotics were detected with LODs ≤10 nM in 1 μL of raw urine and plasma (which was extracted and diluted), and six were detected at ≤500 pM. Sensitivity was increased by a factor of ~3-fold compared to analyses without SPE front-end separation (box on Fig. 1c) and the r^2^ values for the standard curves were >98% for most chemicals, indicating consistent linear responses. Detailed information for the calibration curves is provided in Supplementary material Table S1. Instrument reproducibility was tested using triplicate analyses, and an average coefficient of variance (CV) of <3% was detected for all concentrations, except at the LOD for each chemical which had CVs of ≤8% due to the lower signals. Additionally, the C18 and graphitic carbon cartridges were sufficient for the analysis of all 20 xenobiotics, resulting in a total sample analysis time of 20 s. Thus, the SPE-IMS-MS analyses provided extremely high-throughput measurements with low intensity variability and a linear concentration response.

### 3.3. Quantitation using SPE-IMS-MS

**Fig. 4:**
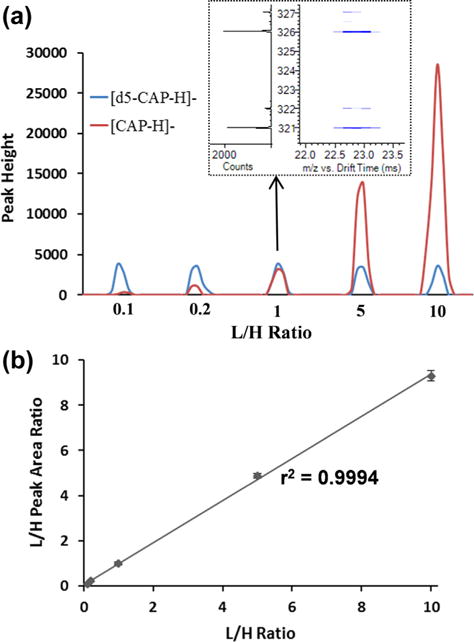
a) The peak intensity values for light and heavy CAP were observed with varying concentration ratios, which correlated with their spiking level (L/H from 0.1 to 10). b) Titration curve created from b) showing high linearity between varying L/H concentration ratios and their responses.

Absolute quantitation in metabolic and exposomic studies is essential for understanding xenobiotic exposure levels and endogenous metabolite changes. The applicability of standard isotope dilution for absolute quantification of chemicals using the SPE-IMS-MS system was evaluated using different light and heavy isotope-labeled chemicals. In one example, chloramphenicol (CAP) was quantified by spiking light (L; unlabeled) and heavy (H; deuterium-labeled) CAP; into human plasma at varying concentrations. CAP is an antibiotic useful in the treatment of bacterial infections, but is only recommended when safer antibiotics cannot be used; thus, understanding how different levels of CAP affect endogenous metabolites is of interest. To demonstrate quantitation of CAP in human plasma samples, heavy CAP, labeled with 5 deuterium atoms (d5-CAP), was spiked into each sample at a fixed concentration of 10 nM, while light (unlabeled) CAP was spiked at concentrations ranging from 1 nM to 100 nM. All samples were then analyzed using SPE-IMS-MS. The peak intensities of the heavy and light CAP molecules at each L/H concentration ratio are presented in Fig. 4a. The titration curve in Fig. 4b shows high linearity (r^2^ > 0.99) between the L/H concentration ratio and the corresponding peak areas, indicating high accuracy of quantitation. All light and heavy pairs studied also had the same IMS drift time, so they were easily identified (Fig. 4a, inset).

### 3.4. Effective separation of isomers and target compounds in complex biofluids

**Fig. 5:**
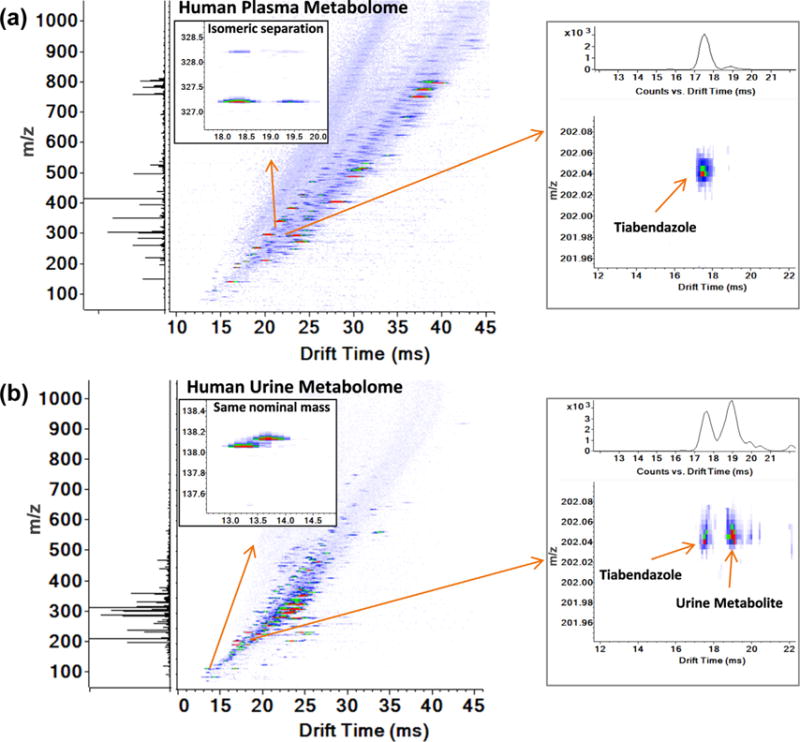
Three-second SPE-IMS-MS analyses of a) human plasma and b) urine extracts with spiked xenobiotics, including 1 nM of tiabendazole. Thousands of features were detected simultaneously in the discovery analyses and isomers were well separated as shown in a) for *m/z* 327.197. Features with the same nominal mass were also distinguished by IMS as shown in b) for *m/z* values of 138.054 and 138.129. Tiabendazole at 1 nM was detected in both human plasma and urine extracts with similar signal intensities.

While SPE significantly improved the measurement sensitivity, IMS separation was also important for resolving multiple isomers in the complex biofluids (*e.g.*, plasma) (Fig. 5a) and separating nominal mass isobars which can be difficult to distinguish by MS/MS when a 0.1 Da selection window is used (Fig. 5b). Fig. 5 demonstrates detection of the pharmaceutical tiabendazole (TAE), which was spiked into human plasma (Fig. 5a) and urine (Fig. 5b), at a concentration of 1 nM. TAE occurred during the 3-s SPE-IMS-MS peak elution time and each sample was analyzed using a 10-s sample-to-sample cycle time to account for cartridge equilibration and sample loading (Fig. 2). While TAE was virtually isolated in plasma, one small molecule in urine had a similar *m/z* value, but was easily separated from TAE with IMS. This allowed for the confident identification of TAE and demonstrated the utility of IMS for reducing false positives in targeted and untargeted analyses of complex biofluids.

### 3.5. Untargeted SPE-IMS-MS analyses by chemical class selection

Untargeted measurements for chemical analytes in complex biological matrices are most successful when interfering substances are effectively removed, target analytes are enriched, and structurally similar classes of compounds and isomers are separated. We tested the effectiveness of high-throughput SPE-IMS-MS analysis using multiple SPE cartridges for analyte enrichment in untargeted studies of 1 μL of raw human urine and plasma, which were extracted and diluted. Initially, five SPE cartridges (i.e., C8, C18, mixed mode, graphitic carbon and HILIC cartridges) representing different packing material chemistries were evaluated with both plasma and urine, and features with S/N ≥5 were extracted using the 4D feature finder in the Agilent IM-MS Browser.

The mixed mode cartridge (which was packed with hydrophobic Imtakt scherzo sm-C18 material and utilized a reversed-phase, cation and anion exchange, and normal phase mode all in the same cartridge) overlapped with many of the hydrophobic identifications obtained for the C8 and C18 cartridges. The mixed mode cartridge also produced the greatest number of features (an average of 1073 features in positive ion mode and 53 in negative ion mode in urine), and thus was selected for the untargeted studies instead of either the C8 or C18 cartridges. The graphitic carbon cartridge preferentially retains hydrophilic chemicals, particularly those with planar structures (such as creatinine and disaccharides). In the graphitic carbon cartridge analysis of urine, an average of 1018 features were detected in positive ion mode and 104 in negative ion mode. Finally, the HILIC cartridge favors smaller hydrophilic molecules (*e.g.*, choline and monosaccharides) and on average detected 371 features in positive ion mode and 58 in negative ion mode. The combination of these three cartridges provided broad coverage of the desired chemical classes and was selected for future untargeted analyses of urine and plasma samples.

**Fig. 6:**
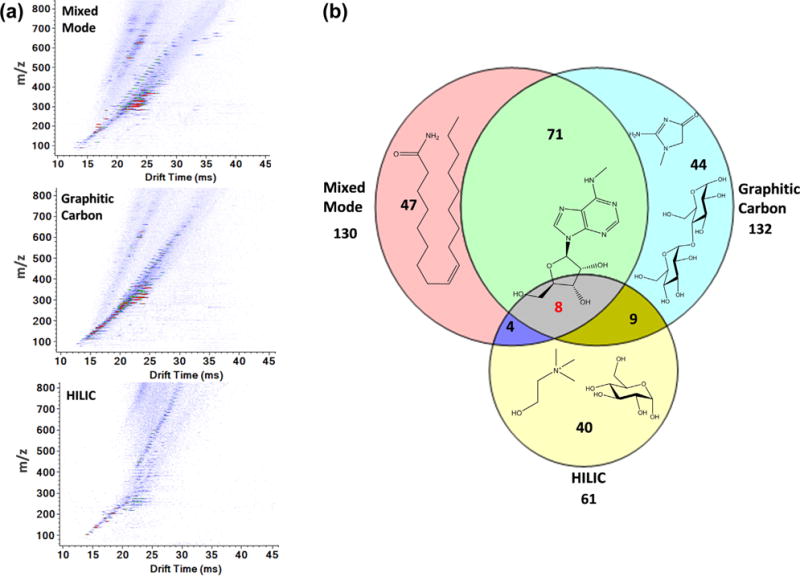
Multiple SPE cartridges were evaluated for global IMS-MS urine analyses of small molecules; a) illustrates the features observed for the mixed mode, graphitic carbon and HILIC cartridges, while b) shows the Venn-Diagram for the unique and overlapping urine features captured by each cartridge. The features are mostly unidentified to date; however, some of the identified features are shown on the Venn-Diagram to illustrate those unique to each cartridge type and the one in common for all three cartridges.

Spectra for urine small molecules retained by the three different SPE cartridges are shown in Fig. 6a. These spectra clearly illustrate that various physico-chemical properties, including hydrophobicity, molecular weight, and structure of the molecules were captured by each cartridge. A Venn diagram of high concentration features detected using the mixed mode, graphitic carbon, and HILIC cartridges is illustrated in Fig. 6b. Only high intensity features (i.e., those with a signal to noise ratio >200) were analyzed in order to understand the binding characteristics of each cartridge. Mixed mode, graphitic carbon, and HILIC cartridges detected 130, 132 and 61 major chemical features using these criteria, and a significant portion of the features were unique to each cartridge, demonstrating the importance of coupling multiple SPE cartridges with IMS-MS.

**Fig. 7:**
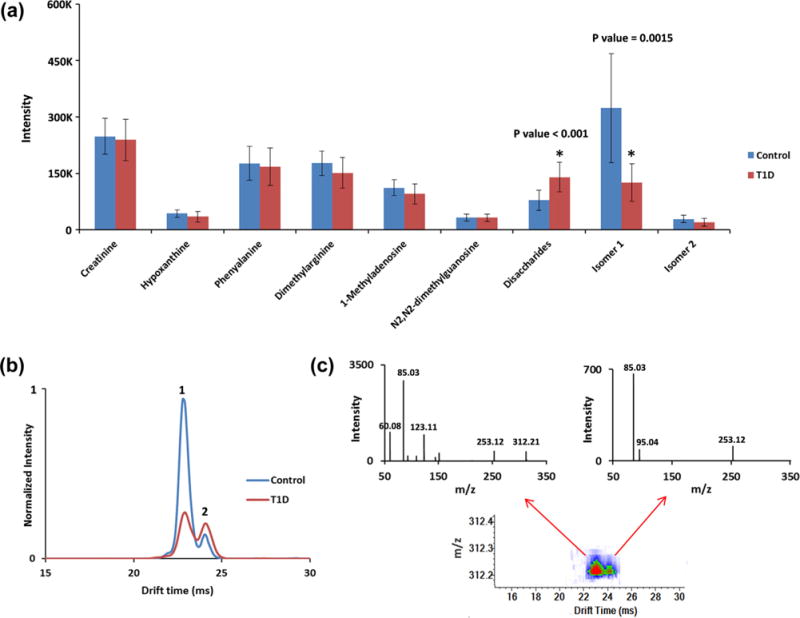
a) Profiles of nine small molecule features from the urine samples for the T1D and control patients. b) Overlaid representative IMS spectra for two features with indistinguishable *m/z* (312.21) for T1D (red trace) and control (blue trace) patients, showing that the IMS separation revealed different feature intensities for the isomers. c) MS/MS spectra for feature 1 and 2 after IMS drift time separation.

The untargeted SPE-IMS-MS analysis strategy using the three SPE cartridges was then applied to study small polar molecules in urine extracted from T1D patients. For this study, urine from 96 individuals (48 T1D patients and 48 sibling controls) was analyzed six times, using each of the three cartridges in both positive and negative ion mode, for 1-min global assessments of each sample. Among the thousands of features detected, 9 example small molecules were extracted and their profiles are displayed in Fig. 7a. Seven of these were identified as endogenous metabolites by matching the *m/z* values with urine metabolites recorded in the Human Metabolome Database (HMDB) [36], with secondary validation via GC–MS. However, the GC–MS and IMS-MS techniques used different ionization techniques (i.e., ESI versus chemical ionization), so these identifications were treated cautiously. Statistical differences for the metabolites observed in the two patient groups were then analyzed using t-tests, and those with p-values lower than 0.05 were selected for further analysis. From these analyses, it was determined that the peak intensity of endogenous metabolites related to energy metabolism (e.g., creatinine), amino acids (e.g., phenylalanine and dimethylarginine), and nucleic acids (e.g., hypoxanthine, 1-methyladenosine and N2, N2-dimethylguanosine) did not significantly change between the T1D patients and their sibling controls. However, the disaccharides (i.e., sucrose and lactose) had statistically significant increases in peak intensity in the T1D patients [37]. Because the resolving power of the IMS used in this study was not able to separate sucrose and lactose, they were both reanalyzed with GC–MS and found to both be significantly higher in the T1D patients. Two major chemical features with indistinguishable *m/z* values (*m/z* = 312.21) were separated by IMS in all of the urine samples, but were not present in any available metabolite databases including the HMDB [36] and METLIN [38], so their identities could not be assigned (Fig. 7b). The feature labeled as “1” had a smaller structure and was detected at an earlier IMS drift time, while the later IMS peak labeled “2” had a larger structure. Feature 1 was significantly lower in intensity for the urine samples from T1D patients, while feature 2 was statistically unchanged between the groups (Fig. 7a). Because these two features were indistinguishable by *m/z*, the patterns were convoluted by MS and MS/MS analysis without IMS separation, causing a combined MS/MS spectrum for the unseparated features. To address this issue, we performed MS/MS of the targeted feature using the SPE-IMS-MS system. The IMS separation allowed the MS/MS spectra for each feature to be analyzed independently since fragmentation is performed after the drift cell (Fig. 7c) [39,40]. Thus, two distinct MS/MS spectra were observed for the two features and, while the different fragmentation patterns will enable identification, the shared fragment ions (m/z = 85.03 and 253.12) suggest the possibility that these two features are structural isomers. However, since these features are not in any current databases their identities are still under investigation.

## 4. Conclusions

In this study, we developed a high-throughput SPE-IMS-MS platform and workflow that allows simultaneous targeted and global assessments of endogenous metabolites and xenobiotics in complex human biofluids. For targeted analyses, highly reproducible and sensitive measurements with LODs in the pM range in biofluids were achieved, while analyte quantitation was obtained with the isotope dilution methodology. Application of multiple SPE cartridges in the global studies allowed enrichment of endogenous metabolites and xenobiotics by chemical class, providing high sensitivity information on their chemical makeup while still maintaining high-throughput (i.e., each sample analysis took <1 min even when studied with three cartridges and positive/negative polarities). In the global urine case-control study for individuals affected by TD1, the IMS separations distinguished isomeric small molecules, aided in their identification with targeted MS/MS measurements, and revealed changing levels of previously unknown isomers associated with TD1. Thus, the rapid throughput, high sensitivity, and quantitative capabilities of the SPE-IMS-MS platform and workflow provide the ability to quickly perform environmental exposure assessment and inpatient screening prior to analysis with lower-throughput methods, if required. Further, the global data collected from the platform could be used for biomarker discovery and metabolic pathway characterization and profiling. We are also exploring several improvements to this platform and workflow for enhanced analyses of nonpolar molecules and more rapid identifications. For example, we are comparing ESI, APCI and APPI sources to determine optimal conditions for studying a large number of polar and nonpolar standards. We are also characterizing thousands of small molecule standards, in triplicate, to determine IMS collisional cross sections and CV values for each. By adding these collision cross sections into large chemical identification libraries which contain exact mass and MS/MS information, rapid feature identifications will be possible. These improvements will allow the SPE-IMS-MS platform and workflow to overcome many challenges in large-scale metabolomic and exposomic analyses and provide a viable path for screening populations and patient cohorts to gain insight into disease processes and human environmental chemical exposure.

## Supplementary Material

Supplemental Information

## Appendix A. Supplementary data

Supplementary data associated with this article can be found, in the online version, at http://dx.doi.org/10.1016/j.clinms.2016.11.002.
