# Supplementary material for: SPE-IMS-MS: An automated platform for sub-sixty second surveillance of endogenous metabolites and xenobiotics in biofluids
Source: Clin Mass Spectrom. 2016 Dec 29;2:1–10. doi: 10.1016/j.clinms.2016.11.002 (PMC5739065; doi:10.1016/j.clinms.2016.11.002)
Supplement: Supplementary data 1 [file mmc1.docx]

**SUPPLEMENTAL TABLE INFORMATION**

Twenty xenobiotic chemical standards (Table S1) were analyzed individually at concentration levels of 10 pM, 100 pM, 1 nM, 10 nM, 100 nM and 1 µM to determine their LOD not in matrix. A mixed stock solution was then prepared using a 99.9/0.1 acetonitrile/formic acid buffer solution and 10 μM of each chemical was used for SPE-IMS-MS method optimization. The mix was added into human plasma extract and human urine extract to achieve final concentration levels of 500 pM, 1 nM, 5 nM, 10 nM, 50 nM and 100 nM for each chemical and the LOD was analyzed in the extracts (Table S1).

**Table S1: Calibration curves for 20 xenobiotics in the human plasma**

| **Detected Ions** | **Calibration Curves** | **R^2^ Values** | **LOD** |
| --- | --- | --- | --- |
| **[Imazaquin+H]^+^** | y = 0.9844x + 0.5892 | 0.9995 | 500 pM |
| **[Hexaconazole+H]^+^** | y = 1.0026x + 0.1707 | 0.9976 | 500 pM |
| **[Thiabendazole+H]^+^** | y = 0.9601x + 0.3117 | 0.9909 | 500 pM |
| **[Metribuzin+H]^+^** | y = 1.0176x - 0.1801 | 0.9954 | 5 nM |
| **[Napropamide+H]^+^** | y = 1.1801x - 1.1967 | 0.9871 | 10 nM |
| **[Flumeturon+H]^+^** | y = 0.7765x + 0.593 | 0.9838 | 10 nM |
| **[Isoxaben+H]^+^** | y = 1.0502x - 0.7122 | 0.9738 | 10 nM |
| **[Fluroxypyr-1-methylheptyl ester+Na]^+^** | y = 1.0122x - 0.5455 | 0.9802 | 10 nM |
| **[Resmethrin+Na]^+^** | y = 0.8232x - 0.0272 | 0.9694 | 10 nM |
| **[Minocycline+H]^+^** | y = 0.8277x - 0.4217 | 0.9852 | 10 nM |
| **[Fenamidone+H]^+^** | y = 0.9607x - 0.701 | 0.9953 | 10 nM |
| **[Fenamiphos+H]^+^** | y = 0.8619x + 0.0479 | 0.9906 | 5 nM |
| **[Fludioxonil-H]^-^** | y = 0.9431x + 0.3504 | 0.9966 | 500 pM |
| **[Oryzalin-H]^-^** | y = 0.8588x + 0.4286 | 0.9982 | 500 pM |
| **[Fluazinam-H]^-^** | y = 0.9545x + 0.3437 | 0.9995 | 500 pM |
| **[Chloramphenicol-H]^-^** | y = 0.8255x - 0.2885 | 0.9598 | 5 nM |
| **[Penoxsulam-H]^-^** | y = 1.0058x - 0.3531 | 0.9989 | 1 nM |
| **[BPSM-H]^-^** | y = 0.8566x - 0.5737 | 0.9991 | 10 nM |
| **[Genistein-H]^-^** | y = 0.6393x + 0.3792 | 0.9963 | 10 nM |
| **[Daidzein-H]^-^** | y = 0.8705x - 0.6366 | 0.9931 | 10 nM |
